# Defining Health Misinformation: Theoretical Concept Analysis

**DOI:** 10.2196/79410

**Published:** 2026-07-28

**Authors:** Johanna Saia Pope, Paula Byrne, Declan Devane, Tina D Purnat, Maura Dowling

**Affiliations:** 1 School of Nursing, Midwifery and Evidence Science College of Medicine, Nursing & Health Sciences Ollscoil na Gaillimhe - University of Galway Galway, Connacht Ireland; 2 Evidence Synthesis Ireland College of Medicine, Nursing & Health Sciences Ollscoil na Gaillimhe – University of Galway Galway, Connacht Ireland; 3 Cochrane Ireland College of Medicine, Nursing & Health Sciences Ollscoil na Gaillimhe – University of Galway Galway, Connacht Ireland; 4 Health Research Board – Trials Methodology Research Network (HRB-TMRN) College of Medicine, Nursing & Health Sciences Ollscoil na Gaillimhe – University of Galway Galway, Connacht Ireland; 5 Harvard TH Chan School of Public Health Harvard University Boston, MA United States

**Keywords:** concept analysis, definition, disinformation, fake news, misinformation

## Abstract

**Background:**

Health misinformation is a serious and growing concern, especially in the era of mass digitalization. However, the term lacks conceptual clarity, reducing our ability to build a reliable, replicable evidence base about how misinformation works and undermining our attempts to develop effective responses. There is, therefore, a need to examine how the term is used and to develop a coherent definition that better reflects people’s information priorities, concerns, and understandings of the concept.

**Objective:**

This study aimed to surface common themes and debates around the concept of “misinformation” in contemporary English-language discourses about health. Specifically, we aimed to examine how people understand the problem of health misinformation (ie, its causes and consequences), how they perceive the relationship between “misinformation” and other problematic information, and any unresolved conceptual tensions.

**Methods:**

We conducted a 3-phase hybrid concept analysis following a framework from Schwartz-Barcott and Kim (2000), comprising (1) a theoretical, literature-based phase, (2) a fieldwork/primary interview phase, and (3) an integrative phase combining findings from the first 2 phases. This paper reports methods and findings from the first phase, an inductive, qualitative literature review and analysis. In this phase, we conducted a systematic search of recent literature on health misinformation (published between 2016 and 2022), selected a stratified random sample, and conducted inductive thematic analysis following the methods outlined by Rodgers (2000). A thematic, narrative summary of the findings is presented herein.

**Results:**

Authors identified rapid technological change, information predators, and cultural issues (eg, social fragmentation, growing epistemic disagreement, and the loss of information authorities) as antecedents to health misinformation. They characterized health misinformation as a scourge, identifiable by its falseness or deceptiveness, the use of persuasive strategies, unscientific subjectivity, and its capacity to disrupt community consensus, and highlighted a broad range of potential consequences for individuals, communities, and social systems. However, there were also major areas of divergence and tension in this literature base. Specifically, there was disagreement about what kinds of problematic content constituted “misinformation,” and how misinformation should be identified or adjudicated, especially in cases of evidentiary uncertainty or legitimate scientific disagreement.

**Conclusions:**

To our knowledge, this is the first review to examine both explicit and implicit definitions of misinformation and to center contemporary usages of the concept. The work identifies several key characteristics of health misinformation, as well as areas where further concept development is needed. The review highlights the need for explicit reporting around the operationalization of the term, implementation of standards for communicating evidentiary uncertainty, values-aligned and co-designed health communications, and continued concept development. Findings from this literature analysis will inform further stakeholder and integrative synthesis, with the goal of developing a more usable, inclusive, and responsive definition of health misinformation.

**International Registered Report Identifier (IRRID):**

RR2-10.12688/hrbopenres.13641.2

## Introduction

Mass digitalization has changed the way people access health information, generating both new opportunities and new risks. For example, digital innovations can help people access and use health information more effectively. They can also play an important supportive role in facilitating shared decision-making and participatory medicine approaches. On the other hand, online platforms and tools have also been heavily implicated in the spread of “misinformation” [1,2].

As awareness of online misinformation has grown, so, too, has interest in its implications for both individual and public health [1]. As a result, there is a growing body of literature aimed at understanding the prevalence [1,2], risk factors [3,4], and consequences [5] of online health misinformation. However, despite growing interest in measuring this concept, it remains ill-defined [1]. When researchers do provide explicit definitions for the term, they tend to reference falsehood [6] and nonconformance with the best available evidence or expert advice [7] as its key characteristics, but there remains significant variation in how the term is actually *used* and *operationalized* across studies [6,7]. The label has been used to condemn a wide variety of information, including conspiracy theories about pandemics [8], vaccine-skeptical beliefs [9], and complementary and alternative health advice [10]. Over time, some argue, the concept has evolved into a catch-all for information or advice deemed problematic or disagreeable, and, perhaps as a result, strategies for deciding which information is “misinformation,” and how misinformation should be measured, remain varied [11].

Inconsistent usage and operationalization of the term may limit communication between researchers; reduce the validity, replicability, or real-world relevance of research findings; and, ultimately, lead to disagreement about which solutions are possible or preferable to reduce its impacts [12]. For these reasons, scholars have called for more work to take stock of how the term is currently understood, identify conceptual ambiguities, and build consensus around the construct [6]. Early work in this area has tended to focus on collating definitions of the term and identifying trends in those definitions [6,7,13]. However, this work has been limited in 2 important ways. First, it has tended to focus on work from within individual disciplines [13] or types of studies [6], limiting our ability to capture the disagreements, tensions, or ambiguities that might be expected to emerge between writers from different fields or with different epistemological orientations. Second, although as many as 85% of research papers do not explicitly report how they have defined “misinformation” in their work, no review has considered implicit understandings of the term [7], meaning that important perspectives and areas of theoretical tension between, or even within, publications may remain obscured.

And, even as researchers struggle to standardize the concept and its measurement, cultural understandings of the term may be changing in response to broader social, cultural, and technological disruptions, such as the COVID-19 pandemic, the rise of social media, and increased access to tools that use artificial intelligence. These disruptions have prompted new kinds of engagement with health information and information technologies, and perceptions of information trustworthiness may be shifting apace. There is, therefore, an urgent need to focus on contemporary discourses around the term, and to analyze how these may be changing as health information technologies—and our relationships to these technologies—continue to evolve.

To address these gaps in understanding of the concept (and hopefully develop a working construct that better reflects people’s current information priorities and challenges), we are undertaking a 3-step, exploratory concept analysis of “health misinformation,” using a framework from Schwartz-Barcott and Kim [14]. In the first step of this work, findings of which are reported herein, we explored how the problem of “health misinformation” is represented in modern English-language research publications, focusing on scholarly work produced between 2016 and late 2022 (when we completed our search). Specifically, we aim to clarify (1) how authors understand the problem of health misinformation (eg, its causes and consequences), (2) the attributes authors associate with “misinformation” and the characteristics they use to identify it, (3) how authors perceive the relationship between “misinformation” and other forms of problematic information (eg, “fake news” or “conspiracy theories”), and (4) any conceptual tensions or disagreements in how misinformation should be defined or conceptualized, including across disciplines, approaches, and time.

Our work in this first literature phase builds on prior reviews by focusing on contemporary usages and delving into implicit understandings, debates, and contradictions in how the term is conceptualized. The approach here is exploratory and inductive: rather than providing a new definition of the term, we aim simply to provide insights into trends and debates in the literature as a basis for future inquiry, including in the second (stakeholder interview) and third (integrative synthesis) phases of the project. In time, we hope that this concept analysis will help ensure a framing of the problem and proposed solutions that are more consistent, timely, meaningful, accessible, and acceptable to the people they aim to support. The findings will be relevant to user experience research, as well as to the design and evaluation of digital interventions to deliver health information, improve health and information literacy, or build resilience to health misinformation.

## Methods

### The Hybrid Approach

We conducted a hybrid concept analysis using a framework from Schwartz-Barcott and Kim [14]. This framework comprises 3 phases: a “theoretical” literature phase, a “fieldwork” interview phase, and a final integrative analysis phase [14]. This paper presents the methods and findings from the first literature phase, which aimed to identify trends and debates in the literature. Subsequent phases will aim to “corroborate and refine” the ideas presented in the literature through consultation with various stakeholders [14], and, eventually, to integrate these into a refined definition of the concept. The results of these subsequent phases will be reported separately.

### Overview of the Literature Phase

This first (theoretical literature) phase of concept analysis was guided by the search, selection, and analysis methods outlined by Rodgers [15]. We selected this approach because it offers a methodological foundation for systematically exploring a concept, while remaining flexible enough to accommodate variability in the concept (eg, across contexts, disciplines, and time) [15,16].

To begin, we searched 6 electronic databases (MEDLINE and PubMed Central via PubMed, Scopus, CINAHL, PsycINFO, and SocINDEX) for papers published between 2016 and 2022 across selected disciplines, in English, on health misinformation. Papers were screened according to preestablished criteria and analyzed using thematic analysis methods outlined by Rodgers [15]. Themes are presented narratively.

### Literature Search and Selection

Rodgers [15] suggested approach to identifying and selecting literature distinguishes itself among concept analysis methods by emphasizing systematic searching and sample representativeness; for instance, Rodgers [15] stresses the need for systematic database searches, rather than the convenience sampling common in other concept analysis methods [15]. Still, as is common in other forms of concept analysis—and in qualitative evidence synthesis methods more broadly—in line with other qualitative synthesis approaches, Rodgers’ [15] approach is also flexible and iterative: it aims to build a sample of literature that, in addition to being representative, is also conducive to deep, inductive thematic analysis and consensus-building [17]. Therefore, in this approach, unlike many others, a single reviewer may use theoretical or purposive considerations to screen papers, extract data, and conduct an inductive thematic analysis [15]. Iterative sampling often continues through the data extraction and analysis phases to ensure that diverse perspectives are represented and important ideas are adequately elaborated [15]. A single-reviewer approach facilitates these returns to the literature base and, later, inductive thematic analysis [15].

However, because our research team comprises several researchers with methodological expertise in evidence synthesis, we considered systematic literature searching and selection to be particularly important to the integrity of the review. Therefore, in building our search plan, in addition to consulting guidance from Rodgers [15], we also referenced Joanna Briggs Institute’s more detailed guidance on scoping review searches, which advises a more rigorous, 3-step approach to literature searching and selection [18].

In the first step, JP, with input from DD and PB, conducted an initial search of PubMed and CINAHL to identify index terms related to health misinformation. Then, assisted by an information specialist, JP developed and implemented a search strategy across 6 bibliographic databases (MEDLINE and PubMed Central via PubMed, Scopus, CINAHL, PsycInfo, and SocINDEX). Full details of this search strategy are available in Multimedia Appendix 1.

Comprehensive database searches were conducted between October and November 2022. After removing duplicates, JP screened titles and abstracts against prespecified eligibility criteria that the full team had agreed on in advance. While not strictly required in Rodgers’ [15] approach to concept analysis, this provided a clear, objective standard for inclusion and exclusion decisions, helping to ensure that the review was objective, transparent, and focused.

Papers were considered eligible for inclusion in the review if they met the following criteria:

1. Focused on the concept of health misinformation. To determine this, we considered whether (1) the “misinformation” discussed in the paper pertained to a human mental or physical health exposure, intervention, condition, or outcome. Human health is complex, and influenced by a wide range of contextual factors. However, in order to facilitate focused analysis of the epistemologies that most directly affect medical and public health decision-making, papers examining information about macrolevel or policy phenomena that exert indirect influences on health, or which lay outside the scope of medical and public health practice (eg, climate change, violence, or criminal justice) were excluded. Papers dealing mainly or exclusively with other types of misinformation (eg, political) were also excluded. (2) Papers made specific reference to “misinformation” or a related term. Concept analysis is traditionally a semantic exercise and often focuses on analyzing the meaning of a specific word [15]. However, Rodgers [15] defines “concepts” more broadly, as “abstractions... expressed in some form”; “not merely the word or expression but the mental cluster that lies behind the word.” Prior work has identified that the term “misinformation” is often used interchangeably with several others [6,7], suggesting that, to examine the “mental cluster” behind this term, we also needed to consider papers that referenced synonymous terms, such as “disinformation,” “fake news,” “alternative facts,” “alternative news,” “post-truths,” “conspiracy theories,” “propaganda,” “false information,” “misleading information,” “unreliable information,” and “bullshit.” We therefore considered any paper that considered one of these terms. However, we did not consider these alternative terms if they were explicitly distinguished from “misinformation” in the work. (3) Health-related misinformation was identified as a focus, theme, or subtheme of the work. To be eligible for inclusion under this subcriterion, papers had to explicitly refer to misinformation (or one of the related terms listed above) in their title, abstract, or keywords, and dedicate substantial attention to addressing these constructs in the text body (eg, by measuring it as an input/outcome or by presenting it as a theme or subtheme of the work). Papers that referred to misinformation only in passing were therefore excluded.
2. Were peer-reviewed and journal-published in one of the following disciplines: medicine and nursing, public health, political science, communication and media studies, information and library sciences, computer science, psychology, sociology, or ethics. These fields were selected to support a comprehensive, multidimensional understanding of the academic concept of health-related misinformation, while maintaining our emphasis on the applications of misinformation in health and health care. Papers published in interdisciplinary journals, or in journals with a very broad disciplinary focus (eg, “science” or “Asia”), were classified according to their academic affiliation or, in the case of authors not affiliated with an academic institution, the author’s disciplinary training as indicated by the subject of their highest degree. Withdrawn/retracted papers and papers that had not yet been peer reviewed were excluded.
3. Were published in or after 2016, and until 2022 (when searches were conducted). Concern about misinformation has been accelerating since the 2010s, and the way people think and talk about it has likely been influenced by recent social and political upheaval (eg, the rise of social media and artificial intelligence, and the COVID-19 pandemic). The year 2016 has been noted as a “turning point” in discourses around this term, and limiting our analysis to discourse published after this time allowed us to focus on how people are thinking and talking about misinformation in the wake of these significant information events, in line with our focus on contemporary conceptualizations of the term [19].
4. Were published in English. Due to the semantic nature of concept analysis, which focuses on understanding the cluster of ideas that underpin a particular expression (such as a particular word) [15], papers had to be written in, or translated into, English, to be eligible under this criterion.

Following screening of titles and abstracts, several thousand articles remained potentially eligible for full-text screening. Rodgers [15] advises that, where there is a significant body of literature on a concept, authors may consider using stratified probability sampling to select papers for inclusion (eg, by discipline, year of publication, or both). Some evolutionary concept analyses choose to stratify by discipline to ensure balanced representation of different disciplines in the final analysis (which facilitates rigorous comparison across disciplines) [15]; however, as the goal of our analysis was to obtain proportional representation of the overall body of literature, we chose instead to stratify all papers that were eligible for inclusion after title and abstract screening by year (2016-2022), and then select a random sample of 10%—the largest number deemed feasible given the resources available—using a random number generator. This stratified random approach helped ensure the representativeness of the sample and was considered preferable to other forms of sample restriction, which would have created more systematic exclusions (eg, by discipline) and therefore undermined the breadth of our final analysis.

Following stratified random sampling by year, JP located full-text articles from the identified literature. When a full-text work was unavailable, the corresponding author was contacted. JP then screened the selected full-text articles.

Following this screening, several hundred papers remained eligible for line-by-line thematic analysis. Because line-by-line analysis of all eligible texts was not feasible, papers were once again sorted by year, and data were collected iteratively for each year in random order until we judged that data saturation had been reached, or until all articles from that year had been analyzed, whichever came first. There are many ways to conceptualize data saturation, but we declared saturation when new data were no longer contributing new codes or refinements [20].

### Data Extraction and Analysis

Following the guidance of Rodgers [15], JP read each article and noted:

1. Attributes the authors assigned to misinformation.
2. Contextual factors (including the discipline from which the authors were writing, the year and journal of each article’s publication, and elements the authors identified as antecedents to, or consequences of, misinformation).
3. Examples of misinformation provided.

Notes were recorded on a standardized form (Multimedia Appendix 2) and analyzed using inductive thematic analysis methods from Rodgers [15], in NVivo software (version 12; Lumivero). Consistent with Rodgers’ [15] concept analysis approach and qualitative synthesis guidance, a single reviewer conducted screening and line-by-line coding, enabling iterative returns to the literature and inductive development of themes. However, this process was supported by regular debriefing and team input to enhance reflexivity and rigor.

Prior to beginning the analysis, JP engaged in a bracketing exercise, labeling prior knowledge of the subject and expectations of the themes that might emerge in the work. JP then conducted line-by-line coding and developed initial themes, and these were reviewed and refined with input from PB and DD. A narrative summary of these themes was drafted, and, subsequently, a second round of review and refinement was conducted by MD and TDP. Final themes and representative quotes were reviewed and agreed upon by all authors.

### Quality Appraisal

Quality appraisal is not recommended in concept analysis [15], where the focus is on the way authors *think* and *talk* about an issue, rather than on the reliability or validity of the empirical findings. We considered it especially important to refrain from judgments about the relative epistemic authority of the voices included in the analysis because of the epistemic nature of debates around this concept.

### Debriefing

Throughout the design and conduct of the review, the primary reviewer (JP) engaged in biweekly debriefings with 2 other members of the research team (DD and PB), during which she was asked to reflect on and discuss methodological decisions and the preconceptions or values underlying them. This helped to create a reflexive research environment.

### Ethical Considerations

This study involved secondary analysis of published literature and therefore did not require human subjects approval.

Reporting was guided by the PRISMA-ScR (Preferred Reporting Items for Systematic reviews and Meta-Analyses extension for Scoping Reviews) checklist (Multimedia Appendix 3).

## Results

### Paper Characteristics

**Figure 1:**
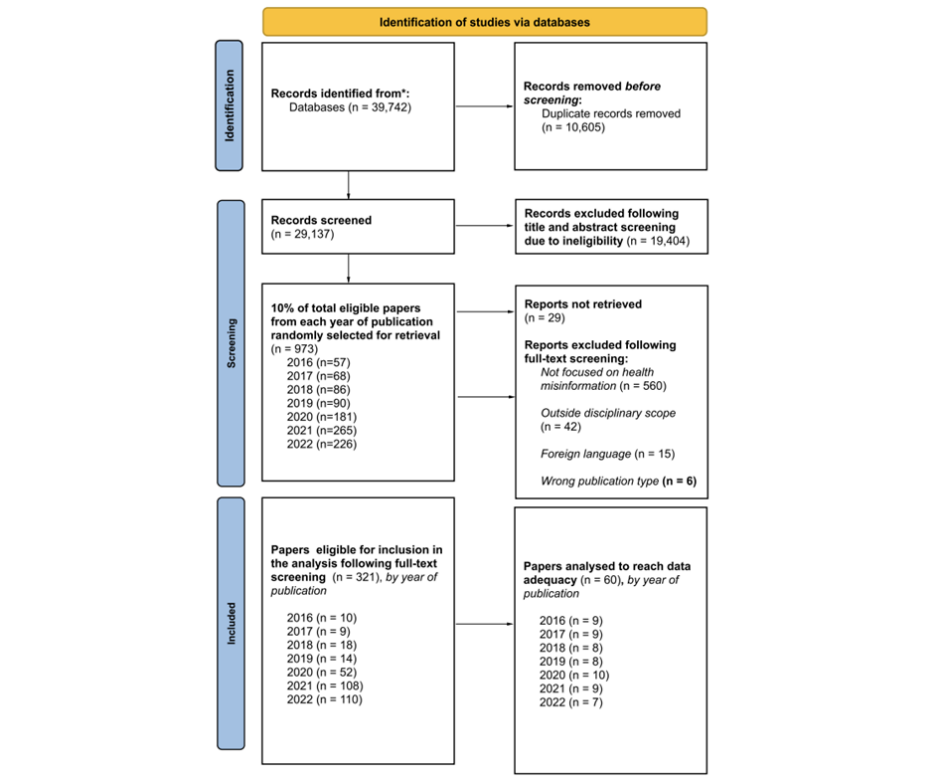
Flow diagram of the identification of studies for review via databases.

Ultimately, we analyzed 60 papers from 9 disciplines. The process of selecting papers for inclusion is documented in Figure 1.

The greatest number of papers included in this analysis were published in 2020 (n=10, 16.67%), and most were from medicine/nursing (n=24, 41.67%) or public health (n=11, 18.33%). A majority (n=38, 63.33%) reported on empirical research, while a significant minority (n=22, 36.67%) were discussions or editorials. Most (n=53, 88.33%) examined health misinformation in the context of a specific health issue (most commonly COVID-19 (n=13, 21.67%) or vaccines (n=11, 18.33%).

Other key characteristics of the literature included in this analysis are provided in Table 1.

**Table 1:** Characteristics of reviewed literature.

Characteristics			n (%)
**Year of publication**			
	2016	9 (15)	
	2017	9 (15)	
	2018	8 (13.33)	
	2019	8 (13.33)	
	2020	10 (16.67)	
	2021	9 (15)	
	2022	7 (11.67)	
**Discipline**			
	Communications	8 (13.33)	
	Computer science	3 (5)	
	Ethics	1 (1.67)	
	Information/library sciences	4 (6.67)	
	Medicine and nursing	24 (41.67)	
	Political science	1 (1.67)	
	Psychology	4 (6.67)	
	Public health	12 (18.33)	
	Sociology	3 (5)	
**Type of literature**			
	Discussion or editorial paper	22 (36.67)	
	Primary empirical analysis	36 (60)	
	Evidence synthesis	1 (1.67)	
	Methods report	1 (1.67)	
**Health topic addressed**			
	Cancer	6 (10)	
	Chronic illness	3 (5)	
	COVID-19	13 (21.67)	
	Dermatology	1 (1.67)	
	Diet and weight management	2 (3.33)	
	Infectious disease and infection control	6 (10)	
	Mental health and substance use	3 (5)	
	Sexual and reproductive health	6 (10)	
	Vaccines	11 (18.33)	
	General health	7 (11.67)	
	Multiple health topics	2 (3.33)	

Despite our focus on English-language literature, the sampled literature included papers authored by researchers from 6 continents and 34 countries. Most were authored by teams from a single continent (n=49, 81.67%). Of these, the majority came from teams based exclusively in North American (n=25, 41.67%) or European (n=15, 25%) institutions. However, 4 (6.67%) papers came from teams based exclusively in Asia, 3 (5%) from teams based exclusively in Oceania, 1 (1.67%) from a team based exclusively in South America, and 1 (1.67%) from a team based exclusively in Africa. An additional 11 (18.33%) papers were produced by teams working across multiple continents, resulting in additional authorship from countries outside of North America and Europe. Nevertheless, authors from North America remained disproportionately represented, with 45% (n=27) of papers listing at least 1 author with an institutional affiliation in the United States, and 8.33% (n=5) listing at least 1 author based in Canada.

More information about the geographic spread of authors is provided in Table 2.

**Table 2:** Geographic spread of paper author teams.

Geographic spread		n (%)
**Single- vs multicountry authorship**		
	Single-country author teams	42 (70)
	Multicountry author teams	18 (30)
**The continent where the author team was based**		
	All Africa	1 (1.67)
	All Asia	4 (6.67)
	All Europe	15 (25)
	All of North America	25 (41.67)
	All Oceania	3 (5)
	All South America	1 (1.67)
	Multiple continents	11 (18.33)
**Distribution of studies by author country (studies with at least 1 author)**		
	United States	27 (45)
	Canada	5 (8.33)
	Italy	6 (10)
	United Kingdom	6 (10)
	Other European countries	19 (31.67)
	Australia/New Zealand	7 (11.67)
	Taiwan	2 (3.33)
	Turkey	2 (3.33)
	Other Asian countries	10 (16.67)
	Brazil	1 (1.67)
	Colombia	1 (1.67)
	Nigeria	1 (1.67)

### Conceptualization of Health Misinformation in the Included Literature

#### Overview

We examined how authors discussed health misinformation to understand (1) factors considered antecedents to health misinformation, (2) attributes associated with health misinformation, and (3) perceived consequences of health misinformation. We then generated themes to describe recurring patterns and debates in the sampled literature. These themes are summarized in Textbox 1 and described in more detail below. It is important to note that these themes reflect conceptual significance rather than numerical predominance.

**Summary of antecedents, attributes, and consequences of health misinformation identified in the examined literature.**

**Antecedents to misinformation**

- Information revolution—and its fallout
- Predators and prey
- Epistemic crisis and the loss of information authorities

**Attributes of misinformation**

- Misinformation as a scourge or contaminant
- Misinformation as falsehood, deception, or persuasive strategy
- Misinformation as noise or disruption

**Consequences of misinformation**

- Consequences for individual well-being (psychological harm, undesirable health choices, and poor health outcomes)
- Consequences for relationships and communities (relationship strain, polarization and division, and undermining of health services)
- Consequences for social systems (threats to social norms and shared understandings; proliferation of alternative perspectives)

#### Antecedents to Health Misinformation

We examined the factors that authors considered important preconditions for health misinformation and developed three themes: (1) information revolution—and its fallout; (2) predators and prey; and (3) epistemic crisis and the loss of information authorities.

#### Information Revolution—and Its Fallout

### Overview

The rise of the internet was characterized as a “revolution” in information-sharing [21] that both democratized the information environment and accelerated the spread of health misinformation. Some authors used the terms “online information” and “misinformation” interchangeably, reflecting a belief that the internet was deeply saturated with misinformation. They expressed ambivalence about engaging with online health information, yet ultimately recognized the necessity of embracing these technologies to deliver accurate health information.

### An Old Problem Gets a Signal Boost

While authors agreed that health misinformation “has always existed” [22], some felt that the internet, and particularly social media, had boosted its spread. Some authors, especially those in communications and computer science, highlighted the role of new technologies (eg, bots and algorithms) in amplifying misinformation. However, they more often argued that people’s interactions with technology fueled the spread of misinformation. They suggested that online, people acted very differently than they would in “real life”: more emotional and ideological, and more prone to engage in groupthink or bully those who expressed opposing views.

Authors described online discussions about health as taking place in polarized “bubbles,” which are guided by their own “illogical logic” [23]. Authors felt these characteristics, and their often short-form, attention-grabbing formats, made online health discourses more compelling than other types of health communication—but also more likely to include misinformation. Health care professionals were cautioned that they might struggle to communicate in these formats:

there is nothing to gain by staff participating in online comment wars... [because] discussion in open online forums rarely follows the kind of reasoned debate clinicians are likely to attempt [24].

Even if staff were exceptionally skilled at “playing” according to digital media’s logic and rules, engaging in online debate... may lead to staff being coopted by the adversarial dynamic that they are trying to avoid [23].

### Ambivalence in the Face of Technological Change

Despite being wary of using technology to engage in health discourse, authors generally agreed that sheer volume made “manual approaches” to correcting misinformation “impractical” [25]. They proposed that, to combat misinformation, it is sometimes necessary to engage in the same practices that spread it. For example, they recommended using artificial intelligence, filters, censors, and the “grammar” of online content to fight misinformation and help their own health messages compete online [21]. Mendonça and Castelfranchi [21], for instance, described how scientists successfully pivoted their communication strategies to gain visibility online during the COVID-19 pandemic:

Visual grammars originated in social networks and online forums were strategically incorporated by some scientists or medical doctors, including memes, TikTok choreographies and narrative genres from YouTube communities.... The aggressive tone of online discussion was also employed. When challenged with conspiracy theories about vaccines, a virologist contested with two words: “Your ass.” The response went viral and the expert ironically claimed that his tweet had become his most cited work [21].

For many authors, embracing digital technologies and platforms represented a necessary adaptation. However, the question of whether and how professionals should engage with online platforms remained unsettled, prompting calls for further research to develop best practices for addressing online misinformation, particularly on social media [26].

#### Predators and Prey

The authors explained how malicious individuals exploited social fragmentation and public vulnerability to spread misinformation.

### Bad Actors and Ulterior Motives

Authors suggested that a significant portion of health misinformation comes from “bad actors” [27] and “content polluters” [28].

Sometimes, these actors were portrayed as “trolls” [29], flooding the internet with mixed messages simply to sow chaos and “[render] the information ecosystem incomprehensible” [27]. More frequently, however, they were characterized as issue advocates, spreading misinformation to influence public attitudes about specific health interventions. Authors, therefore, perceived misinformation as clustering around a few controversial health issues (eg, vaccines, contraceptives, or non-Western medicine). Authors in this study were especially concerned about messaging from “antivaxxers,” who were characterized as belonging to an ideological fringe, but some suggested that more “official” organizations, such as pharmaceutical companies, could spread misinformation, too. For example, Crosswell and Porter [30] highlight how Merck spread “propaganda” to promote sales of the human papillomavirus vaccine. Still others focused on health misinformation spread by elected leaders.

### Vulnerability in a Hostile Climate

Authors described an information culture characterized by alienation, polarization, and mistrust, exacerbated by growing social inequity and the trauma of the COVID-19 pandemic. They expressed concern that the public lacked the skills and resources to protect themselves against manipulation from bad actors. While they sometimes disagreed about who was most vulnerable, many felt interventions to enhance mental health and resilience were essential for safeguarding people against misinformation in this hostile environment.

#### Epistemic Crisis and the Loss of Information Authorities

### Overview

Authors observed an increasing mistrust of “official” sources and explained how, as traditional gatekeepers’ influence has diminished, the public has sought out other, often less scientific, sources for health information. They expressed concern that this shift might lead people to encounter and accept health misinformation, while also acknowledging the limitations of science in establishing truth.

### The Decline of the Neutral Gatekeeper

The authors suggested that even sources traditionally viewed as reliable and neutral gatekeepers of information (eg, mainstream media, scientists, and health care professionals) had become implicated in spreading misinformation. For instance, they suggested that reporters might share unverified information because they lacked the skills or time to vet it properly, or because they were more interested in attracting “hits” or “likes” than in ensuring information accuracy. Likewise, they suggested that peer-reviewers and journal editors, traditionally regarded as bulwarks against academic misinformation, were increasingly “abdicating their responsibility” as scientific gatekeepers to save time, profit from publication fees, or uphold existing health care policies [31].

With gatekeepers’ integrity under scrutiny, some authors went so far as to question the existence of a “neutral” or “reliable” gatekeeper. These authors argued that, even in the absence of misconduct or malice, reporting on health issues is inevitably influenced by subjective factors such as social position, cultural attitudes, and personal risk perceptions—biases that can undermine their ability to serve as objective arbiters of truth. Nonetheless, the authors lamented what they viewed as the “death of expertise” [21], which they believed would create chaos in the information environment and enable unreliable or nonpreferred sources to gain greater influence over what people believe.

### Diverging Epistemic Values (or How Should We Know What’s True?)

Authors described a public increasingly aware of the limitations of information authorities and scientific processes, and more likely to seek out information from a broader range of sources, including nonscientific ones. While some authors felt that this might improve holistic health, they were more often concerned that, as information became democratized and old information hierarchies dissolved, it was becoming much harder to adjudicate debates about what is true. Some characterized this situation as an “epistemic crisis”:

The recognition that other forms of knowledge matter and that narratives and testimonies are relevant to problem-solving has nurtured side-effects, such as the depreciation of science and dangerous forms of equivalence that transform everything into a matter of opinion [21].

Some authors condemned what they viewed as the public’s growing relativism and declining interest in objective facts. For instance, Ruppel et al [32], observing women in a reproductive health support group, remarked on the “dangerous” trend of treating all health advice as equally valid, including advice based on anecdote rather than “established resources”:

Though emotions occasionally ran high, most disputes resolved when posters agreed to disagree rather than when views consolidated around a particular position.... Note Poster 2’s statement in the excerpt above that “anything is possible,” and Poster 1’s final statement: “[The medical consensus] doesn’t make either one of us wrong. It means our opinions differ.” This lens could be at the root of posters’ reliance on personal experience over established resources. More significantly, since many medical questions do have both correct and incorrect answers, the perception that all posters’ medical opinions were equally worthwhile could be potentially dangerous [32].

The idea that all health messaging is “equally worthwhile” was regarded by some as a symptom of dangerous posttruthism [32]. In particular, authors expressed frustration that the public seemed to place equal value on scientific and nonscientific information, especially when nonscientific information was influenced by emotions, cultural values, or social factors.

Although some authors raised concerns about the limits of scientific evidence in resolving debates about what is considered “true” and what is deemed “misinformation,” especially in situations of evidentiary uncertainty or where there were genuine, subjective disagreements regarding the risks associated with a specific disease or treatment, most ultimately felt that scientific evidence was the best available arbiter of truth:

You can look at the horizon and see that it is “virtually” flat, but the fact is that the Earth is not flat! We do not “agree to disagree” and suggest that it is a hemisphere (half flat and half round)... [where t]here is overwhelmingly strong scientific evidence [32].

#### Attributes of Health Misinformation

#### Overview

We examined the characteristics that researchers associate with health misinformation and identified several common themes. First, there was consensus among the authors in this study that misinformation is a serious problem. Second, they generally agreed that, for information to be considered misinformation, it needed to be either false or misleading, according to “the science.” Beyond its falsehood or divergence from the scientific consensus, however, the authors in this study also distinguished misinformation by its aggressive stance toward, and capacity to disrupt, community cohesion.

#### Misinformation as a Scourge or Contaminant

Health misinformation was recognized as a significant challenge with potentially immediate and deadly consequences. More difficult to identify than political information [33], health misinformation was seen as an insidious, evolving threat that could appear in various forms, affect all kinds of people, and shape public perceptions even after it was debunked.

Some spoke of misinformation in medicalized terms. They compared misinformation to a contaminant or “disease” [25], which had “virulent effects” [34] and could be as “contagious as a virus itself” [35]. Left unchecked, they cautioned, misinformation could cascade into an “infodemic” (an “information pandemic”). For these authors, then, health misinformation was a serious pathology that could undermine both individual and community health [36]:

infodemic induced confidence crisis and distrust in authorities, science communities, governments and institutions can... become a serious threat to public and global mental health as another kind of virus... [the] “pandemic is a physical exam of the social body, and never has public trust been put to a bigger test.” [36]

#### Misinformation as Falsehood, Deception, or Persuasive Strategy

##### Misinformation as Falsehood or Deception

Most explicit definitions of health misinformation focus on its “absolute factual accuracy” [25], or on its intention to persuade or deceive. Some authors applied the term to any verifiably false health information (eg, that COVID-19 is caused by 5G), while others reserved the label for intentionally misleading information.

##### Misinformation as a Persuasive Strategy

Some authors warned that any information with a persuasive slant or bias should be approached with caution, and some labeled this type of information as “misinformation” or “propaganda,” regardless of its absolute accuracy. Persuasive information was not always viewed negatively, however. Stanchevici [24], for example, describes a piece of “propaganda” aimed at raising funds to help eradicate tuberculosis in Moldova:

[The] images depict abject poverty and misery: tiny apartments with battered furniture, dilapidated Soviet-era hospitals, dark prison cells overcrowded by emaciated inmates, an old decrepit trolleybus, poorly dressed and sickly people. The video alternates slides containing information about MDR-TB [multidrug-resistant tuberculosis], photo images, and interviews with some of the people in the photos telling their sad stories of living with TB [tuberculosis]... [over] a melancholic Moldovan folk tune [24].

This information was labeled as “propaganda” not because it was false or malicious (tuberculosis was indeed a significant issue in Moldova, and its eradication was a public health priority), but because it used emotions to achieve a purpose (ie, to persuade donors to support a cause). Generally, however, this type of misinformation was characterized by fearmongering or maliciously distorted risks. For example, Swartzendruber et al [37] provide the following description of health misinformation aimed at discouraging use of contraceptives:

Sites commonly mentioned unknown health effects of [emergency contraception], which we considered to be misleading given lack of evidence to warrant concern about these effects. For example... several sites emphasized unknown risks among “women who have not started their periods” and “postmenopausal women,” individuals not even at risk of pregnancy 37.

Other persuasive strategies, such as “mimicking the language of mainstream news” [38] or appealing to emotions, preexisting beliefs, and other cultural values, were also noted. For instance, Moran et al [39] described how antivaccine misinformation often included strong appeals to values:

Anti-vaccine websites contain a considerable amount of misinformation... [and] values such as choice, freedom, and individuality were linked to anti-vaccine beliefs... One page, for example, linked vaccine refusal to freedom, stating “We need to fight... for OUR COUNTRY, OUR CONSTITUTION, OUR FREEDOM, and OUR CHILDREN !!!” [39]

#### Misinformation as Unscience and Subjective Belief

To determine whether health information was false, most authors recommended consulting scientific evidence or guidelines from reputable institutions. Some did so explicitly: for instance, in their study of vaccine misinformation in parenting groups on Facebook, Bradshaw et al [40] operationalized vaccine misinformation as any “information presented that appeared to contradict standard childhood vaccination recommendations as issued by the CDC [Centers for Disease Control and Prevention] and American Academy of Pediatrics.”

Scientific evidence and sources were frequently cited as objective arbiters of truth, and, conversely, information that was not objective was perceived as “misinformation.” For example, in a study of parents who reported “no intent” to vaccinate their adolescent daughters for human papillomavirus, Cheruvu et al [41] asked participants to select their reasons for declining, including “vaccine misinformation.” Among the items described as “misinformation” were opinion statements that could not be empirically tested (eg, their beliefs that the vaccine was “Not Needed or Not Necessary,” that their daughters were not an “Appropriate Age” to receive it, or that they had “Increased Sexually [sic] Activity Concern[s]”) [41].

Some authors argued that any information based on experiences or opinions, rather than evidence, could be considered misinformation. This included personal experiences, which were often labeled as misinformation even if they contained no definitive falsehood or deception. In their report on misinformation in antivaccine websites, Moran et al [39] provide the following example:

One page... reported a mother’s story about her child: “He was hospitalized two days after the shot and he was running a fever of 103. And he was so hot that the nurse that was standing there could feel the heat radiating off his body [sic].” Jeremy’s mother Lynn said. For the last 29 years, Jeremy has not spoken an intelligible word [39].

#### Misinformation as Noise or Disruption

For many authors, however, the issue of misinformation extended beyond individual falsehoods, pseudoscience, opinions, or experiences; it was also characterized by norm violations, challenges to authority, and the promotion of “antisystem ideas” such as “no to the new world order” or simply “resistance to comply with measures dictated by governments” [29].

Authors suggested health misinformation and conspiracy theories were often produced and perpetuated by other social issues, such as class (eg, “There is a cure for AIDS, but it’s being withheld from the poor”) or racial tensions (eg, “AIDS is a form of genocide against blacks”) [42,43]. Sometimes, health misinformation was also seen as reflecting “larger sociopolitical and ideological debates” or “culture wars” [23]. However, health misinformation could also be used to exacerbate these tensions, and indeed some argued that this was often its primary intention [44].

Misinformation can foster an atmosphere of panic and discrimination.... Early in the AIDS response, President Reagan missed a vital opportunity to address discrimination against children with AIDS, refusing to state clearly that he would send his child to a school with other children living with AIDS.... Similarly, President Trump regularly referred to SARS‐CoV‐2 as the ‘Chinese virus’ early in the COVID‐19 response, turning attention away from the crucial task of controlling the pandemic and potentially instigating racial tensions [44].

Consequently, some authors defined health misinformation not only by its falseness but also by its disregard for, or hostility toward, community cohesion and the “common good.”

#### Potential Consequences of Health Misinformation

Authors in this study suggested that health misinformation could negatively impact individuals, communities, and social systems.

##### Consequences for Individual Well-Being

Authors emphasized that health misinformation could inflict psychological harm by increasing individuals’ uncertainty, anxiety, or fear. Some mentioned the potential for health misinformation to cause new information-related disorders, such as “headline stress disorder” [45]. In addition, they suggested that misinformation could lead people to adopt attitudes, perceptions, or behaviors inconsistent with established health guidance.

Many authors also expressed concern that exposure to misinformation might make people more likely to develop attitudes they perceived as “wrong” or otherwise problematic (eg, belief in alternative medicine or vaccine hesitancy) or that it could distort their perception of health risks. This could, in turn, lead to undesirable health choices, including delaying needed care, engaging in risky behaviors, or simply failing to “obey the medical recommendations” [46]. At the end of this chain of effects, they warned, health misinformation could potentially lead to poorer health outcomes and increased mortality, making it “literally... a matter of life and death” for some [33,47].

##### Consequences for Relationships and Communities

Authors in this study suggested that health misinformation could also affect individuals at the community level, causing strain on personal relationships and within community groups. Many referred to health misinformation as a source of division and polarization, leading, in turn, to increased distrust of others, and particularly of health officials. They highlighted that health misinformation could damage relationships between patients and providers and, more broadly, undermine public health initiatives.

##### Consequences for Social Systems

In addition to these individual and relational risks, health misinformation was perceived as carrying threats to established norms, with the potential to “advance alternative agendas” [27] and to challenge shared understandings of health issues.

Some authors worried that, over time, this could erode the public’s belief in the possibility of shared understandings. For these authors, misinformation posed a risk not only because it affected how people understood health issues or which health behaviors they engaged in, but also because it could reduce their “belief in truth itself,” “challenging its social capacity to function as a normative principle” [21]:

*misinformation anddisinformation... saturate and drastically distort the discourses and narratives of the global public sphere. Such conditions render societal convergence on agreed sets of facts, widespread agreement on what constitutes reality, and functional collective sense-making, all increasingly challenging and unlikely to be realized.* [27]

#### Debates and Ambivalence in Definitions of Health Misinformation

Above, we have described common themes in this literature. Below, we highlight several areas of disagreement and tension.

##### The Promise and Perils of Teaching Reliability Cues

Authors acknowledged that even experts may struggle to identify misinformation, for example, because accurate assessment often requires technical knowledge, or because some misinformation is designed to be “distractingly plausible” [48] to nonexpert audiences. Some authors, therefore, suggested that general audiences could use reliability cues (eg, source credibility, tone, academic rigor, or alignment with recommendations from major health organizations) to make informed judgments about whether something might be misinformation. Others cautioned that these cues are not always reliable indicators, since some health misinformation originates from credible or “expert” sources.

##### Information Used Right (and Wrong)

While most explicit definitions of health misinformation focused on its falsehood or ability to mislead, many authors also used the concept to refer to information that is problematic in other ways. As noted above, the term was often used to describe undesirable opinions or beliefs, regardless of whether they were technically or scientifically “true.” In other cases, the term “misinformation” was applied to describe information that is lacking in other ways, even if it was not strictly false: for example, information that is insufficient, irrelevant, or otherwise not fit for purpose. This includes information that lacks nuance, clarity, or guidance about how to act. It also encompassed information that, while technically accurate, was not tailored to an individual’s health situation or context. Ruppel et al [32] give an example of this type of misinformation:

While some information offered was technically correct, the people offering it had limited information on the specifics of the recipient’s health, and were rarely trained in adapting data to an individual situation. During one discussion, a poster listed fertility medications and dosages that her partner had tried, and commenters suggested what they would recommend trying in a future cycle based on their own experiences. This advice was given without consideration for varying causes of infertility, or an understanding of the risks and benefits of different treatment options[32].

Moderation and verification were therefore deemed essential to ensure that the information people received was complete and relevant to their situations. However, the best strategies for achieving this remained unclear, particularly in cases of evidentiary uncertainty or where legitimate disagreements existed regarding the risks of a disease or treatment for an individual.

## Discussion

### Principal Findings

The authors of these papers identified rapid technological change, information predators, and social fragmentation as major antecedents to health misinformation. They characterized health misinformation as a sort of information disease, and defined it, variously, as information that was false, deceptive, persuasive, unscientific, subjective, or threatening to community cohesion. While the problem of health misinformation was framed primarily around its consequences for physical and mental health, many authors were also very concerned about the potential of health misinformation to disrupt consensus about health issues. Crucially, they were also concerned about its potential to undermine public faith in the value of such consensus. However, the study also identified several tensions in definitions of health misinformation, and, especially, a lack of clarity about the characteristics that make it problematic, and disagreement about how information quality should be adjudicated in situations where scientific consensus is limited.

Below, we explore how the findings from this work align with prior work to define the term.

### Comparison to Prior Work

Our concept analysis builds on prior work to clarify the definition of misinformation by probing the implicit meanings, assumptions, and values latent in how the term is used. Our very broad search for literature and inclusive approach to selecting articles facilitated the identification of philosophical tensions in how the term is conceptualized, as well as inconsistencies in its use. It is, to our knowledge, the first study to empirically explore these debates. In addition, by focusing on recent literature, we were able to surface emerging debates and unresolved conceptual tensions, both within individual works and between them.

Below, we reflect further on how our findings align with prior work to define the term.

### What Is Health Misinformation?

In line with previous findings [6,7], most authors characterized health misinformation as falsehood or divergence from scientific evidence. At the same time, they acknowledged the difficulty in judging these characteristics, particularly in situations where evidence may be lacking or conflicting, or where information conveys subjective risk assessments. In these instances, some suggested deferring to expert or institutional consensus. Others, however, cautioned that such sources may not always be reliable. This suggests a need to clarify how misinformation should be assessed in cases of scientific uncertainty or disagreement.

In addition, some forms of health misinformation identified in this literature could not be assessed against scientific evidence because they represented individual stories, experiences, emotions, or value judgments, rather than testable facts. Authors in this study found this kind of information problematic because it was produced and sustained by nonscientific epistemologies, and they often suggested interventions to shift the public’s epistemic values to better align with scientific ones. However, some have suggested that it may be misguided to draw bright lines between “scientific” and “value-laden” information, given the significant body of theoretical work (and emerging empirical evidence) suggesting that scientific decision-making is often motivated by value-based considerations, and concerns that dismissing nonempirical information as “misinformation” may alienate audiences [49-52]. We suggest another limitation of this approach: that it fails to acknowledge health promotion efforts that rely on storytelling or other narrative formats to convey important and accurate health information, including, significantly, some content produced by scientific and health professionals adapting to the formats (or “grammars”) of social media [21].

Furthermore, some authors were concerned about the limitations of current scientific approaches in providing actionable information for the public and in distinguishing misinformation from true information. Some recommended that, especially when scientific evidence is inconclusive, professionals engaged in health communication should offer information aligned with audiences’ values and priorities. At the same time, authors often characterized expressions of these values and priorities as problematic, or even “misinformation,” suggesting unresolved ambivalence about the role of lay values in health communications.

Finally, the review surfaced concerns about the role of digital technologies in the provision of health information. Many authors perceived mass digitalization as a threat to the development and maintenance of a healthy information ecosystem, and some treated “online health information” and “health misinformation” as interchangeable concepts. This skeptical framing of digital health information—as inherently untrustworthy—may be problematic to the extent that it does not recognize the valuable role accurate digital health communications can play in providing information to the public.

Given these tensions in the construct, we suggest the need for more serious empirical analysis of the epistemological values that inform how people access and use health information, and for building a construct of health misinformation that better reflects the public’s current information-seeking behaviors, priorities, and values. We will commence this work in the second fieldwork phase of this concept analysis; findings from this stage will be reported separately.

### What Is the Scope of the Problem Considered to Be?

Authors overwhelmingly felt that health misinformation was a major and growing issue for public health and health care. Some identified specific ways that health misinformation could negatively impact health or health care provision. More commonly, however, they framed health misinformation as a problem in and of itself, focusing on its characteristics and content rather than tying it to behavioral, health, or public health objectives. This perhaps reflects scientific uncertainty about the effects of health misinformation, which have been inconsistent across studies and measurement approaches, and may vary based on contextual factors [53].

Some authors used explicitly medicalized language to describe health misinformation, likening it to an infection or a pandemic. We believe this pathologized framing of health misinformation is misguided: first, because patients who feel that health officials do not share their values may be less likely to discuss their information habits, or engage in shared decision-making, with health care providers [54,55], and, second, because it encourages mechanistic and medicalized “treatments” that may not adequately consider contextual factors, including the broader social, structural, and informational inputs that shape how people access and use information [12]. We believe that this framing should be shifted to better acknowledge the broader factors that influence information access, use, and trust [12].

A key first step could be establishing greater consensus about which information dysfunctions qualify as “misinformation,” and which should be designated differently. In this literature, authors used the term “misinformation” to describe a wide range of problematic or objectionable content, including misconceptions, intentional disinformation, conspiracy theories, and “fake news,” as well as persuasive information, statements containing value judgments or emotional charges, undesirable opinions, personal stories, and otherwise true information presented out of context.

This aligns with findings from a recent review by Nan et al [11], who, noting this variability, suggested that the term “health misinformation” might serve as an umbrella term for a broad spectrum of challenging or problematic health information. That approach has the advantage of allowing us to tackle the issue of health misinformation from various angles and levels of analysis. However, it also blurs the distinctions between different information problems, reducing analytic precision and limiting the development of tailored solutions [12]. In addition, it may exacerbate problems of interdisciplinary miscommunication and threaten the replicability of research findings. Therefore, we recommend that, while efforts to define this concept are ongoing, researchers studying health misinformation should not presume that the term will be universally understood and should clearly state how they have conceptualized and operationalized the problem in their individual work and how this conceptualization may have shaped the findings.

### Reflections on Positionality

Our research team included researchers with both topic expertise in health promotion and infodemic management and methodological expertise in evidence synthesis. These perspectives shaped our approach to study design, data collection, and analysis in several ways.

First, the inclusion of researchers with applied public health experience shaped our line of inquiry, and, especially, the decision to examine both “misinformation” and related constructs in our analysis. The decision to take a wide view of misinformation likely shaped the themes that developed and the theoretical tensions we captured. During the research process, we identified additional priorities for practice (such as identifying differences or disagreements in how the problem was framed), and this analytical choice is also likely reflected in the findings.

Second, our status as relative “insiders” in examining how misinformation is discussed among research colleagues may have limited our ability to challenge assumptions or fallacies in the discourse around this subject. In particular, because we were already familiar with the literature on this concept and had read prior reviews examining its definitions, we expected many definitions of health misinformation to be anchored in scientific epistemologies and evidence. To help manage and dilute this insider perspective, we cast a wide disciplinary net and examined literature outside the immediate health sciences, including political science, information science, and sociology. This approach allowed us to branch out into unfamiliar literature, and we were, at times, surprised by what we found there. We expect that our perspectives will be further broadened by fieldwork in the second phase of this work, which examines conceptualizations of misinformation in contexts beyond academic or research publishing.

More generally, it is worth noting that all researchers entered the study with a significant interest in the interaction between evidence and decision-making, and especially, the values people use to evaluate health claims when evidence is uncertain. These are problems that we will continue to explore in the next interview phase of this work.

Finally, while our methodological approach was initially based primarily on concept analysis methods from Rodgers [15], we also drew heavily on methods from other, more systematic forms of evidence synthesis, for example, in our use and reporting of a 3-step search plan from Joanna Briggs Institute [18], preplanned screening criteria, and a standardized form to extract data from papers [17]. This, we feel, strengthened the rigor and transparency of our overall methodological approach, while preserving the inductive qualitative orientation that makes concept analysis such a strong complement to other, more positivist forms of reviewing.

### Strengths and Limitations

Our study has several limitations. First, while our review used—and, we believe, benefited from—a systematic approach to searching, screening, and selecting literature, we did not strictly adhere to systematic review methods, and took several steps to reduce the number of papers screened and analyzed to a manageable size. For example, we only considered papers that referenced health misinformation (or a prespecified related term) in their titles, abstracts, or keywords. While we believe this helped ensure that the papers we considered placed health misinformation at the center of their analysis, and therefore enabled a richer, deeper inductive analysis, this decision also limited the comprehensiveness of the review.

Second, following screening of all nonduplicate titles and abstracts identified in our search (n=29,137), 9733 papers remained eligible for full-text screening. Recognizing the need for some form of “quantity control,” but cautious about further restricting our sample in a way that might unnecessarily bias or limit the usefulness of the findings, we undertook an extended discussion to develop a sampling frame.

During this discussion, which included significant input from experts in evidence synthesis on our team, we considered several factors. First, we identified the need to reduce the number of citations to a size that would allow for rigorous eligibility screening and line-by-line, inductive analysis. Then, we weighed the potential risks of reducing the sample size by introducing other exclusion criteria. Ultimately, we agreed that this would create unwanted and systematic differences between the citations that passed on to full-text and those that did not, undermining our ability to produce a balanced review, engage with diverse literature, draw out conflicting epistemologies/ways of thinking about misinformation, and, ultimately, to progress our understanding of the concept beyond prior work. These latter considerations were regarded as especially important given the identified need to move beyond mechanistic or single-level understandings of the concept, and to integrate diverse problematizations of information dysfunctions in health spaces (eg, across research approaches, levels of analysis, discipline, or time) [12]. Finally, we consulted guidance from Rodgers [15], whose methods underpin the rest of this review, and best practices in qualitative evidence synthesis, where breadth, depth, and diversity are considered more relevant considerations than comprehensiveness, and sampling approaches may be iterative and/or theory-driven to support the identification of an appropriate sample for analysis [17].

This decision reduced our sample to a “cross-section,” and rendered our review noncomprehensive. However, we do not see this as an impediment to rigor, because our aim was to explore, qualitatively, commonalities and disagreements in how people understand the concept of health misinformation, and how they differentiate it from other forms of information [15]. In line with the philosophy underpinning evolutionary concept analysis, this was intended to help us build a foundation for future inquiry [15], rather than to exhaustively map published definitions of the term [6,7], or summarize the breadth of evidence about its effects [3,53]. Similar sampling approaches have been successfully used in previous qualitative evidence syntheses (eg, Ames et al [56]); and the widely-used concept analysis methods from Rodgers [15], on whose guidance this work is based, explicitly endorse stratified random—or even purposive—sampling of relevant literature when necessary to conduct a rich analysis. However, our approach goes beyond methodological expectations in concept analysis by using strict, preestablished criteria to identify relevant papers, prioritizing the randomness (and therefore the representativeness) of the papers eventually selected for inclusion in the analysis, and transparently reporting the number of papers located in the search and the number included/excluded at each stage [15].

Our sample was restricted in several other ways as well. First, while our included publications from teams working across the world, we only considered literature published in English. This was an intentional decision and reflects the discursive focus of concept analysis, which invites the researcher to analyze the ideas represented by a particular word (or, in this case, cluster of words) [15]. On the other hand, it also reduced our ability to capture diverse ways of speaking about information issues, including in other languages. The findings may, therefore, be less relevant or applicable in non–English-speaking contexts.

Similarly, our focus on preidentified terms used to describe unreliable information (such as “misinformation” and “fake news”) naturally narrowed our lens and may have limited our ability to consider work that addressed other related constructs, such as public trust or science denialism. Given the construct ambiguities identified in this work, future reviews might adopt a more expansive approach to literature identification and selection, if feasible. However, given the significant body of work identified here, other restrictions (eg, by discipline, type of work, etc) would likely be necessary.

In addition to being language-restricted, our review focuses on recent literature drawn from a relatively short publication period (2016-2022). We chose to focus on this period because it is widely agreed that, at least since the 2010s and particularly since 2016, we have been undergoing significant political, public health, and technological disruption, which have likely changed the way people approach information-seeking and think about information credibility [19,57-59]. Focusing on contemporary literature allowed us to capture contemporary themes and tensions. Significantly, it also facilitated exploration of shifts and disagreements that may have emerged during the COVID-19 pandemic, a period when discourse on this topic accelerated rapidly [60]. Despite this restriction, we expect that the themes presented in this analysis were likely very significantly influenced by seminal works published before 2016 (as, by nature of being “seminal,” these publications would be expected to have influenced subsequent approaches to thinking about and measuring the concept). We therefore suggest that this work be read as an expansion on prior work tracing the origins and traditional definitions of the term.

Despite the restrictions noted here, this work remains, to our knowledge, the most comprehensive and interdisciplinary screening and analysis of literature on this subject to date, and the only one to explore implicit meanings and debates.

### Directions for Future Research

While we briefly noted conceptual tensions across disciplines regarding intentions, persuasive strategies, and scientific authority, explicitly comparing how these vary between disciplines was beyond our current scope. Future research could systematically examine these disciplinary differences to illuminate how they shape the conceptualization of misinformation and the framing of potential solutions. This could also help inform how specific disciplinary framings shape the range of solutions offered and inform more targeted interdisciplinary interventions to combat health misinformation.

Similarly, there is a need to examine conceptual tensions and differences in the application of the concept between different stakeholders, including laypeople. We have begun this work in phase 2 of our concept analysis (currently underway), which explores usages of the term “misinformation” in a variety of nonacademic contexts, and examines how the themes identified in academic work are manifest (or not) in these settings.

### Conclusion

This review identifies key patterns in academic understandings of “health misinformation.” It is, to our knowledge, the first study to take a qualitative approach to examining this concept, and the first to examine how the concept is used across disciplines and study types. This approach allowed us to capture both consensus and tension in this concept. Furthermore, it provides a line-by-line inductive analysis of how the term is used in recent literature—a novel approach which allowed us to move beyond inventorying definitions to analyzing implicit meaning-making and debates in the application of the concept. Finally, by focusing on recent publications, we were able to center contemporary understandings of the term, identify evolutions and developments in how the concept is understood, and identify emerging debates and tensions around the concept’s application.

We hope that our paper offers a snapshot of how the concept “health misinformation” has been applied across disciplines in recent years. By identifying definitions and drivers of health misinformation, the review also provides an empirically grounded agenda that can be refined into an actionable taxonomy, including in the second and third phases of this work. Ultimately, we hope that these findings will contribute to developing a more standardized and people-centered approach to measurement, evaluation, and reporting in misinformation research, and to the development of interventions that more adequately address people’s information concerns. We therefore see the work as having potential implications for a wide range of professionals working on topics related to misinformation, including those engaged in user experience research and those involved in the planning, delivery, or evaluation of digital health communications and information literacy interventions.

In the meantime, our synthesis highlights 3 immediate leverage points for action. First, transparency standards—detailing the evidential status and degree of uncertainty—could help practitioners communicate without overpromising certainty [61]. Second, values-aligned messaging suggests co-designing health content with communities rather than broadcasting expert verdicts [62]. Third, ecosystem interventions—such as search algorithm adjustments or prebunking prompts—address structural drivers and may complement person-level health literacy efforts, if approached with awareness of their limitations [62,63]. We suggest that evaluating such multilevel strategies should be a priority for the third phase of this project and broader research on health misinformation.

## Appendix

Multimedia Appendix 1 Literature search strategy.

Multimedia Appendix 2 Standardized data extraction form.

Multimedia Appendix 3 PRISMA-ScR checklist.

## Abbreviations

PRISMA-ScR: Preferred Reporting Items for Systematic Reviews and Meta-Analyses extension for Scoping Reviews

## Data Availability

The data supporting this study consist of bibliographic records and extracted qualitative codes. The search strategies are provided in Multimedia Appendix 1, and the standardized data extraction form is provided in Multimedia Appendix 2. Extracted, deidentified data, and coding summaries are available from the corresponding author upon reasonable request. The codebook is available on Open Science Framework [64].
