# Supplementary material for: Defining Health Misinformation: Theoretical Concept Analysis
Source: J Med Internet Res. 2026 Jul 28;28:e79410. doi: 10.2196/79410 (PMC13462726; doi:10.2196/79410)
Supplement: Supplementary file 2 [file jmir_v28i1e79410_app1.docx]

**APPENDIX 1:**

**LITERATURE SEARCH STRATEGY**

1. **CINAHL search query**
   (TI health?related OR AB health?related OR MW health OR TI medical OR AB medical OR TI medication* OR AB medication* OR TI well?being OR AB well?being OR MW science OR TI science* OR AB science* OR TI scientific OR AB scientific OR MW covid-19 OR TI pandemic* OR AB pandemic* OR MW vaccine OR TI vaccine* OR AB vaccine* OR TI vaccination* OR AB vaccination* OR MW immunization)

**AND**

(MW misinformation OR MW disinformation OR TI misinfo OR AB misinfo OR TI disinfo OR AB disinfo OR TI “alternative fact” OR AB “alternative fact” OR TI “conspiracy theor*” OR AB conspiracy theor*” OR TI “fake news” OR AB “fake news” OR TI “false news” OR AB “false news” OR TI infodemic* OR AB infodemic* OR TI truth OR AB truth OR TI falsehood* OR AB falsehood* OR TI “false information” OR AB “false information” OR TI “misleading information” OR AB “misleading information” OR TI “unreliable information” OR AB “unreliable information” OR TI “incorrect information” OR AB “incorrect information” OR TI “untrustworthy information” OR AB “untrustworthy information” OR TI “inaccurate information” OR AB “inaccurate information” OR TI “poor quality information” OR AB “poor quality information OR MW quackery OR TI gossip OR AB gossip OR TI hoax* OR AB hoax* OR MW deception OR TI “urban legend*” OR AB “urban legend*” OR TI myth* OR AB myth* OR TI fallac* OR AB fallac* OR TI miscommunication* OR AB miscommunication* OR TI rumo?r OR AB rumo?r OR TI propaganda OR AB propaganda)

***Search limits:***
**Limiters -** Published Date: 20160101-20220931; Peer Reviewed
**Search modes -** Boolean/Phrase

***Updating search:*** Email alerts

2. **PsycInfo search query**

(MJ health OR TI health OR AB health OR TI medical OR AB medical OR TI medication OR AB medication OR MJ medical sciences OR TI well?being OR AB well?being OR MJ well being OR MJ sciences OR TI science* OR AB science* OR MJ science communication OR TI scientific OR AB scientific OR MJ covid-19 OR TI covid-19 OR AB covid-19 OR MJ pandemics OR TI pandemic* OR AB pandemic* OR MJ vaccination OR TI vaccination* OR AB vaccination* OR TI vaccine* OR AB vaccine* OR MJ vaccination attitudes)

AND

(MJ misinformation OR TI misinformation OR AB misinformation OR TI disinformation OR AB disinformation OR TI misinfo OR AB misinfo OR TI disinfo OR AB disinfo OR TI "alternative fact*" OR AB "alternative fact*" OR TI "conspiracy theor*" OR AB "conspiracy theor*" OR TI "fake news" OR AB "fake news" OR TI "false news" OR AB "false news" OR MJ infodemic OR TI infodemic OR AB infodemic OR MJ truth OR TI truth OR AB truth OR TI falsehood OR AB falsehood OR TI false NEAR/3 information OR AB false NEAR/3 information OR TI misleading NEAR/3 information OR AB misleading NEAR/3 information OR TI unreliable NEAR/3 information OR AB unreliable NEAR/3 information OR TI incorrect NEAR/3 information OR AB incorrect NEAR/3 information OR TI untrustworthy NEAR/3 information OR AB untrustworthy NEAR/3 information OR MJ deception OR TI deception OR AB deception OR TI "urban legend*" OR AB "urban legend*" OR MJ myths OR TI myth* OR AB myth* OR TI fallac* OR AB fallac* OR TI miscommunication* OR AB miscommunication* OR TI inaccurate NEAR/3 information OR AB inaccurate NEAR/3 information OR TI "poor quality" NEAR/3 information OR AB "poor quality" NEAR/3 information OR TI quackery OR AB quackery OR MJ gossip OR TI gossip OR AB gossip OR TI hoax* OR AB hoax* OR TI rumo?r OR AB rumo?r OR MJ propaganda OR TI propaganda OR AB propaganda)

***Search limits:***

**Limiters -** Publication Year: 2016-2022; Published Date: 20160101-20220931; Peer Reviewed; Exclude Dissertations
**Search modes -** Boolean/Phrase

***Updating search:***

Email alerts

3. **PubMed Central search query**

(Health[tiab] OR medical [tiab] OR medication*[tiab] OR wellbeing[tiab] OR "well-being"[tiab] OR science[mesh] OR science*[tiab] OR scientific[tiab] OR "covid-19"[mesh] OR covid[tiab] OR pandemic*[mesh] OR pandemic*[tiab] OR vaccine[mesh] OR vaccination[mesh] OR vaccine*[tiab] OR vaccination*[tiab] OR immunization[mesh])

**AND**

(misinformation[tiab] OR disinformation[mesh] OR misinfo[tiab] OR disinfo[tiab] OR "alternative fact*"[tiab] OR "conspiracy theor*"[tiab] OR "fake news"[tiab] OR "false news"[tiab] OR infodemic[mesh] OR infodemic* [tiab] OR truth[tiab] OR falsehood*[tiab] OR "false information"[tiab] OR "misleading information"[tiab] OR "unreliable information"[tiab] OR "incorrect information"[tiab] OR "inaccurate information"[tiab] OR "poor quality information"[tiab] OR quackery[mesh] OR gossip[tiab] OR hoax*[tiab] OR deception[mesh] OR "urban legend*"[tiab] OR myth*[tiab] OR fallac*[tiab] OR miscommunication*[tiab] OR rumor[tiab] OR rumour[tiab] OR "untrustworthy information”[tiab] OR propaganda[mesh] OR “false belief*” [tiab])

***Search limits:***

**Limiters -** Publication Year: 2016-2022

***Updating search:***

Email alerts

4. **Scopus search query**

((TITLE-ABS-KEY(health OR medical OR medication* OR well?being OR science* OR scientific OR covid-19 OR covid OR pandemic* OR vaccine* OR vaccination* OR immuni?ation) AND PUBYEAR > 2015 AND PUBYEAR < 2023) AND (TITLE-ABS-KEY(misinformation OR disinformation OR misinfo OR disinfo OR "alternative fact*" OR "conspiracy theor*" OR "fake news" OR (false PRE/3 news) OR infodemic* OR truth OR falsehood* OR (false PRE/3 information) OR (misleading PRE/3 information) OR (unreliable PRE/3 information) OR (incorrect PRE/3 information) OR (untrustworthy PRE/3 information) OR (inaccurate PRE/3 information) OR ("poor quality" PRE/3 information) OR quackery OR gossip OR hoax* OR deception OR "urban legend*" OR myth* OR fallac* OR miscommunication* OR rumo?r OR propaganda) AND PUBYEAR > 2015 AND PUBYEAR < 2023) AND ( LIMIT-TO ( DOCTYPE,"ar" ) ) )

***Changes in search strategy, vs. protocol:***

Syntax corrected after search yielded error: no results. Terms containing “PRE/3” were edited to contain parentheses.

***Search limits:***

**Limiters** - Publication Year: 2016-2022 ; Document Type: Article

***Updating search:***

Email alerts

5. **socINDEX search query**
   (SU health OR TI health OR AB health OR KW health OR TI medical OR AB medical OR KW medical OR TI medication OR AB medication OR KW medication OR SU medical sciences OR TI well?being OR AB well?being OR KW well?being OR SU well-being OR TI science* OR AB science* OR KW science* OR TI scientific OR AB scientific OR KW scientific OR SU covid-19 pandemic OR TI covid-19 OR AB covid-19 OR KW covid-19 OR TI pandemic* OR AB pandemic* OR KW pandemic* OR SU vaccination OR TI vaccination* OR AB vaccination* OR KW vaccination* OR TI vaccine* OR AB vaccine* OR KW vaccine* OR SU immunization)

**AND**

(SU misinformation OR TI misinformation OR AB misinformation OR KW misinformation OR TI disinformation OR AB disinformation OR KW disinformation OR TI misinfo OR AB misinfo OR KW misinfo OR TI disinfo OR AB disinfo OR TI “alternative fact*” OR AB “alternative fact*” OR KW “alternative fact*” OR TI “conspiracy theor*” OR AB “conspiracy theor*” OR KW “conspiracy theor*” OR SU FAKE news OR TI “fake news” OR AB “fake news” OR KW “fake news” OR TI “false news” OR AB “false news” OR KW “false news” OR TI infodemic OR AB infodemic OR KW infodemic OR SU TRUTHFULNESS & falsehood OR TI truth OR AB truth OR KW truth OR TI falsehood OR AB falsehood OR KW falsehood OR TI false NEAR/3 information OR AB false NEAR/3 information OR KW false NEAR/3 information OR TI misleading NEAR/3 information OR AB misleading NEAR/3 information OR KW misleading NEAR/3 information OR TI unreliable NEAR/3 information OR AB unreliable NEAR/3 information OR KW unreliable NEAR/3 information OR TI incorrect NEAR/3 information OR AB incorrect NEAR/3 information OR TI untrustworthy NEAR/3 information OR AB untrustworthy NEAR/3 information OR KW untrustworthy NEAR/3 information OR TI inaccurate NEAR/3 information OR AB inaccurate NEAR/3 information OR KW inaccurate NEAR/3 information OR TI “poor quality” NEAR/3 information OR AB “poor quality NEAR/3 information OR KW “poor quality” NEAR/3 information OR TI quackery OR AB quackery OR KW quackery OR SU gossip OR TI gossip OR AB gossip OR SU HOAXES OR TI hoax* OR AB hoax* OR KW hoax* OR SU deception OR TI deception OR AB deception OR KW deception SU URBAN folklore OR TI “urban legend*” OR AB “urban legend*” OR KW “urban legend*” OR SU MYTHOLOGY OR TI myth* OR AB myth* OR KW myth* OR TI fallac* OR AB fallac* OR KW fallac* OR SU MISCOMMUNICATION OR TI miscommunication* OR AB miscommunication* OR KW miscommunication* OR SU RUMOR OR TI rumo?r OR AB rumo?r OR KW rumo?r OR SU PROPAGANDA OR TI propaganda OR AB propaganda OR KW propaganda)

**Search limits:
Limiters** - Peer Reviewed; Date of Publication: 20160101-20220931
**Search modes** - Boolean/Phrase

**Updating search:**
Email alerts
