# Supplementary material for: Defining Health Misinformation: Theoretical Concept Analysis
Source: J Med Internet Res. 2026 Jul 28;28:e79410. doi: 10.2196/79410 (PMC13462726; doi:10.2196/79410)
Supplement: Supplementary file 3 [file jmir_v28i1e79410_app2.docx]

**Appendix 2:**

**Standard Data Extraction Form**

1. **Paper Title:**
2. **Authors’ Names:**
3. **Year of Publication:**
4. **Discipline:**
5. **Study design:**
6. **Health topics addressed:**
7. **Primary word used to identify unreliable information:**
8. **Characteristics of misinformation identified by authors:**
9. **Indicators used to rate information reliability (if applicable):**
10. **Identified antecedents to misinformation:**
11. **Identified consequences of misinformation:**
12. **Surrogate terms:**
13. **Related concepts:**
14. **Provided examples of misinformation:**
15. **What audience/information consumers were the authors interested in?**
16. **What (personal or population) characteristics did the authors identify as contributing to misinformation belief?**
17. **Strategies proposed to address misinformation:**
